# Thermal effect of a 445 nm diode laser on five dental implant systems: an in vitro study

**DOI:** 10.1038/s41598-021-99709-8

**Published:** 2021-10-11

**Authors:** Herbert Deppe, Markus Ahrens, Alexandra V. Behr, Christina Marr, Anton Sculean, Petra Mela, Lucas M. Ritschl

**Affiliations:** 1 Department of Oral and Maxillofacial Surgery, School of Medicine, Technical University of Munich, Ismaninger Straße 22, 81675 Munich, Germany; 2 Department of Mechanical Engineering and Munich School of BioEngineering, Chair of Medical Materials and Implants, Technical University of Munich, Boltzmannstraße 15, 85748 Garching, Germany; 3 Department of Periodontology, University of Berne, Freiburgstraße 7, 3010 Berne, Switzerland

**Keywords:** Medical research, Experimental models of disease, Dental diseases

## Abstract

The purpose of this in vitro study was to assess the thermal effect of the 445 nm diode laser on five dental implant systems. In an ailing implant protocol, five commercial dental implant systems were subjected to 445 nm diode laser energy at different wattages [W], exposure times, and modes (continuous wave [CW] vs. pulsed and contact vs. non-contact) of laser beam delivery. Scanning electron microscopy (SEM) allowed the evaluation of irradiated implant surfaces. A total of 2880 temperature response curves were recorded. The 445 nm wavelength caused temperature increases of more than 10 °C at or above the 0.8 W power level working in CW mode for 5 s and in pulsed mode at 3 W for 20 s with 10% duty cycle. Highest rises in temperature were seen in the Straumann Pure ceramic implant, lowest in the Ankylos system. SEM analysis revealed no surface alteration in all systems in non-contact mode. The applied laser is not inherently safe for the decontamination of ailing implants. From the results of this study it was concluded that different dental implant materials and geometries show different temperature response curves when subjected to 445 nm diode laser energy. Clinicians ought to be aware of this. Therefore, manufacturers of laser devices should provide implant-specific laser parameters for the decontamination process. However, both laser irradiation systems can prevent harmful rises in temperature and surface alteration when used at moderate laser parameters.

## Introduction

Over the past years, dental implants have become an integral part in oral rehabilitation. Therefore, a continually increasing number of patients are treated with dental implants in Europe and worldwide^1^. As a consequence, an increasing number of complications may be expected over the long term and, among these, inflammatory peri implant diseases^2^.

Peri-implant mucositis was characterized as a well-defined inflammatory lesion lateral to the junctional/pocket epithelium with an infiltrate rich in vascular structures, plasma cells, and lymphocytes by the World Workshop on the Classification of Periodontal and Peri-Implant Diseases and Conditions in 2017. Peri-implantitis was defined as a pathological condition which is plaque-associated and occurrs in tissues around dental implants. This goes along with an inflammation in the peri-implant mucosa and subsequent progressive loss of supporting bone^3^. According to current epidemiologic studies, 2.6–4.0 million patients are at risk of developing peri-implantitis worldwide every year^1,4^. However, successful treatment options for this inflammatory disease are few^1,5^.

Due to the results of a recent review of the literature, anti-infective treatment protocols for the elimination of bacterial microbiota included a pretreatment phase (nonsurgical supramucosal biofilm removal) followed by decontamination of the implant surface using various techniques^6^. Scarano and coworkers^2^ divided these detoxifying techniques as follows: mechanical detoxification using a plastic curet, rubber cups, ultrasonic scaler, or air-powder abrasives; chemical detoxification using citric acid, chlorhexidine, tetracycline, hydrogen peroxide, or stannous fluoride; and laser-based treatments. Nevertheless, mechanical and chemical techniques are not inherently safe with respect to the implant surface chemistry and topography^7^. The excellent long-term results of dental implants are attributable to a superficial titanium dioxide layer (TiO_2_), which prevents corrosion of the metal in the body and is, therefore a prerequisite for undisturbed osseointegration^8^. Accordingly, techniques used for the decontamination process need to respect chemical and topographical integrity of the implant surface to allow re-osseointegration. However, several present decontamination techniques may exert adverse effects to the contaminated implant surface during the detoxification process. It is known from the literature that use of dental curettes and air-powder abrasives does not result in either sterile or isotonic sites^9^. In addition, chemical substances for detoxification such as citric or phosphoric acids may be harmful to the healing process. Moreover, mechanical and chemical methods may lead to implant surface changes viewed on scanning electron microscopy, without effectively detoxifying the surfaces^2,10^. Furthermore, some of these methods are not innocuous; in fact, sudden death caused by embolism has been reported following the infracrestal application of air-powder abrasives^11^.

Therefore, to overcome the shortcomings of conventional decontamination, dental lasers were suggested for the treatment of peri-implant infections, based on its advantages such as easy handling, and hemostatic and bactericidal effects against periodontopathic pathogens while reducing bleeding, swelling, and pain^2^. Several laser-based methods are highly effective in sterilization and detoxification^2^. Some of the studies have dealt with the influence of laser irradiation on the endosseous titanium surface^12,13^, with re-osseointegration^14,15^ and with laser modification of titanium for improved cell adhesion^16^. Moreover, different laser wavelengths are utilized in dental implantology for second stage surgery and biofilm removal during peri-implantitis therapy^17,18^.

However, not all laser systems available in dentistry are of value for this purpose. Appropriate laser wavelengths for implant surface decontamination ought to be well absorbed in water to vaporize the watery biofilm and the inflammatory granulation tissue. In addition, the wavelengths used should inactivate cytotoxic lipopolysaccharides. Moreover, absorption characteristics of implant materials need to be taken into consideration. If laser wavelengths are absorbed in the material of the fixture to a significant extent, laser energy may cause detrimental thermal effects on the implant and, in consequence, on the surrounding soft and hard tissues. Accumulated laser heat on the implant surface can cause chemical reactions, converting TiO_2_ to compounds that are less resistant to microabrasion and chemical aggression in the body. Accordingly, Bida reported that neodymium:yttrium-aluminumgarnet (Nd-YAG) laser irradiation on an implant collar, using a power setting of 3.0 W at 20 pulses per second, resulted in slight pitting of the surface^19^. Moreover, Block and coworkers noted that the potential exists for Nd-YAG laser irradiation to melt and even to remove the surface layer from plasma-coated titanium implants^20^.

On the other hand, it has been shown in the beagle dog model that CO_2_-laser–assisted decontamination enables sterilization of exposed implant surfaces and re-osseointegration^15^. The most likely explanation for this is that carbon dioxide laser energy is not absorbed to any significant extent by metallic surfaces. This reduces the potential risk for damage to the implant and thermal injury to the surrounding tissues. Additionally, due to its excellent absorption in water carbon dioxide laser irradiation has an important effect for sterilization purposes and, therefore consecutively in the biofilm as well^15,21,22^.

With progressive development in laser technology, diode lasers are becoming increasingly important. They are reported to be associated with multiple applications, ease of use, relative safety, and low cost^17,18,23,24^. However, diode lasers could also cause overheating of the implant surface recent in vitro studies^17,24,25^. In this context, if the critical temperature increase of 10 °C is exceeded, the vitality of surrounding bony and soft tissue structures could be affected^26^.

On the other hand, a recent review of in vivo and in vitro studies analyzed the antibacterial effect of diode lasers with different wavelengths (810 nm, 940 nm, and 980 nm) and evaluated their effects on implant surfaces for peri-implantitis treatment^27^. The authors described no critical increase in temperature by using a 810 nm diode laser, which was also associated with complete or almost complete elimination of bacteria from the implant surface as adjunctive tool in combination with conventional therapy^14,27^.

A new diode laser device with a blue wavelength of 445 nm was recently introduced into dentistry^28^. At this wavelength, antimicrobial effects showed superior results compared to those of infrared diode laser radiation and promoted an effective disinfection of contaminated tissue areas. This concomitant effect provides clinically relevant support for the use of a 445 nm laser for surgical indications^28^. However, comparatively little is known about the thermal effect of the 445 nm diode laser energy on dental implants or the surrounding bone when this device is used for the decontamination process. Therefore, the purpose of this study was to assess in vitro the thermal effects of this laser wavelength on five different commercial dental implant systems.

## Results

### Thermal effects

Table 1 shows the temperature increases, recorded at the cervical thermocouple, when the implants were subjected to laser energy. Due to the total of 2880 single measurements, the presentation of the results is focused on parameter combinations which had caused maximum clinically acceptable rises in temperature (∆T) of about 10 °C, including (1) continuous wave (CW) mode and pulsed mode of laser beam delivery and (2) low and high power output (0.8 to 3.0 W).

**Table 1:** Maximal cervical temperature increases (∆T [°C], mean ± SD) following 445 nm laser irradiation in five dental implant systems.

	Mode	NobelReplace	Xive S plus	Ankylos	Straumann pure ceramic	Straumann SLA Bone Level Roxolid
CW mode (0.8 W, 5 s) H = 10,526 J cm^−2^	Contact	8.42 (± 1.13)	8.83 (± 0.52)	7.42 (± 0.88)	9.50 (± 0.5)	7.83 (± 1.26)
	Non-contact	7.50 (± 0.50)	7.67 (± 0.52)	6.00 (± 0.25)	8.17 (± 0.76)	5.92 (± 0.38)
pulsed mode DC 10% (3 W, 20 s) H = 15,789 J cm^−2^	Contact	9.83 (± 0.80)	7.50 (± 0.50)	7.33 (± 0.76)	9.75 (± 1.39)	7.75 (± 1.09)
	Non-contact	7.92 (± 0.88)	6.33 (± 0.38)	5.57 (± 0.76)	7.58 (± 0.80)	6.25 (± 0.25)
pulsed mode DC 50% (1 W, 10 s) H = 13,157 J cm^−2^	Contact	9.17 (± 2.25)	8.58 (± 0.52)	8.08 (± 0.80)	9.42 (± 0.63)	8.67 (± 1.13)
	Non-contact	8.67 (± 0.80)	7.08 (± 0.52)	6.50 (± 0.90)	8.50 (± 0.5)	7.75 (± 0.5)

Both continuous wave and pulsed modes were applied in contact and non-contact mode.

*DC* duty cycle (%), *CW* continuous wave.

**Figure 1:**
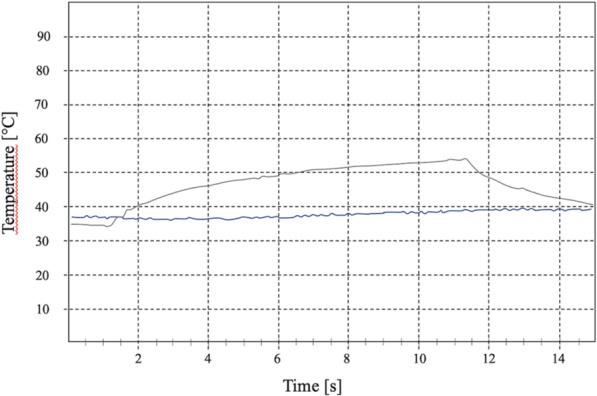
Temperature response curve for the NobelReplace Tapered Groovy RP 4.3 × 10 mm implant when subjected to 445 nm laser irradiation at 2 W for 10 s in continuous wave and non-contact mode. The blue line indicates the temperature at the apical recording point.

The 445 nm wavelength caused remarkable temperature increases above the 0.8 W power level when working in CW mode, both in contact and non-contact mode of laser beam delivery (Fluence H = 10,526 J cm^−2^) (Table 1). At this power level and at an exposure time of only 5 s, ∆T increased between 5.92 °C (± 0.38) (Straumann SLA Bone Level Roxolid, non-contact) and 9.50 °C (± 0.5) (Straumann Pure ceramic, contact), in contact mode of laser beam delivery (Fluence H = 10,526 J cm^−2^). Higher levels of power or longer laser light exposures were not acceptable with respect to the temperature increase from a clinical point of view (Fig. 1).

**Figure 2:**
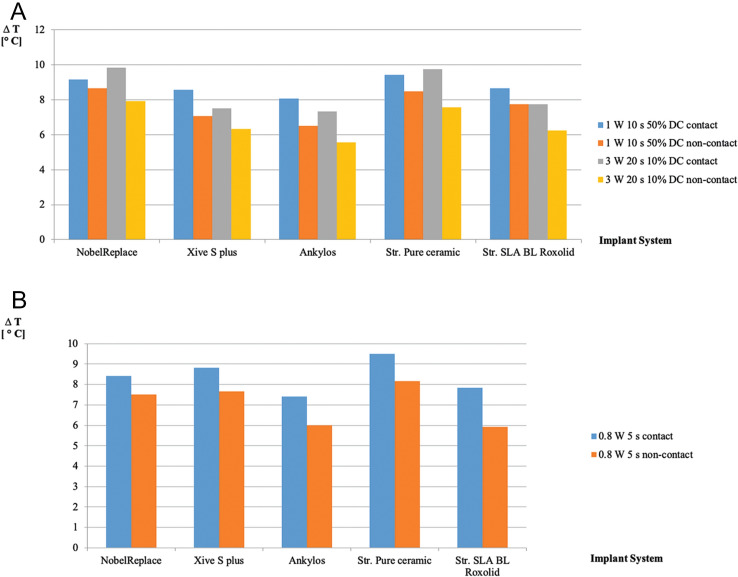
Temperature response curves for the increase in cervical interface temperature (∆T [°C]) as a result of different wattages (0.8 W), exposure times (5 s) and modes in five commercial dental implant systems (NobelReplace, Xive S plus, Ankylos, Straumann Pure ceramic and Straumann SLA Bone Level [BL] Roxolid): (**A**) Wattage 1–3 W, exposure times 10 s and 20 s in pulsed mode. (**B**) Wattage 0.8 W, exposure time 5 s in continuous wave mode.

When working with the pulsed mode, the 10% duty cycle allowed the application of the maximum power output of 3 W even for 20 s, both in contact and non-contact irradiation (H = 15,789 J cm^−2^), resulting in a maximum of ∆T of 9.83 °C (± 0.80), contact (NobelReplace). Lowest rise of temperature was seen in the Ankylos implant with 5.57 °C (± 0.76), non-contact mode (Table 1 and Fig. 2 A).

In pulsed mode with the 50% duty cycle, the combination of parameters (1 W, 10 s) caused similar results. When using these parameters (H = 13,157 J cm^-2^), clinically acceptable rises in temperature of maximally ∆T 9.42 °C (± 0.63) were seen (Straumann Pure ceramic, contact) (Table 1). Lowest rise of temperature was seen, again, in the Ankylos implant with 6.50 °C (± 0.90), non-contact mode (Table 1 and Fig. 2A).

All cervical temperature response curves showed that, when working with the 50% duty cycle in contact mode, an exposure time of 5 s was enough to exceed the 10 °C level at power levels above 1.6 W (11.1 °C). When working with the 50% duty cycle in non-contact mode at the latter levels of power, the 10 °C level was reached after 10 s (9.8 °C). Temperature response curves demonstrated that a power of 3.0 W resulted in a temperature increase of more than 7 °C within 2 to 3 s when the 50% duty cycle was used in the non-contact mode of laser application.

It seems to be of notable interest that, when implants were exposed to CW laser energy (Fig. 2B), the five different implant systems did not respond completely similar to laser energy. Highest rises in temperature were seen in the Straumann Pure ceramic implant system, both in contact and non-contact mode. In contrast, lowest rises were found in the Ankylos system in contact mode of laser beam delivery and in the Straumann SLA Bone Level Roxolid implant in non-contact. Increases were statistically significantly different between implants, both in contact and non-contact mode of laser beam delivery (Table 2).

**Table 2:** Statistical analysis of the maximal and minimal thermal effects (∆T [°C], mean ± SD) of 445 nm laser irradiation on cervical interface temperature in five dental implant systems.

Laser-parameters	Minimum	Maximum	*p*-value
**CW mode (0.8 W, 5 s)**			
Contact	Ankylos 7.42 (± 0.88)	Straumann Pure ceramic 9.50 (± 0.5)	0.02*
Non-contact	Straumann Roxolid 5.92 (± 0.38)	Straumann Pure ceramic 8.17 (± 0.76)	0.01*
**Pulsed mode DC 10% (3 W, 20 s)**			
Contact	Ankylos 7.33 (± 0.76)	Straumann Pure ceramic 9.75 (± 1.39)	0.06
Non-contact	Ankylos 5.57 (± 0.76)	Nobel Replace 7.92 (± 0.88)	0.03*
**Pulsed mode DC 50% (1 W, 10 s)**			
Contact	Ankylos 8.08 (± 0.80)	Straumann Pure ceramic 9.42 (± 0.63)	0.09
Non-contact	Ankylos 6.50 (± 0.90)	NobelReplace 8.67 (± 0.80)	0.04*

*Statistical significance on the 5% level.

Similarly, the five different implant systems did not respond completely similarly to laser energy when exposed to pulsed mode (Fig. 2A). The Straumann Pure ceramic implant showed highest increases among all systems following contact exposure to 1.0 W laser power for 10 s with 50% duty cycle and following 3.0 W for 20 s with 10% duty cycle. However, differences failed to reach statistical significance (Table 2 and Fig. 2A). Moreover, it can be seen from Fig. 2 A that, among the four titanium implants, the Ankylos system showed the lowest rise in temperature when exposed to pulsed laser energy, both following the application of 1.0 W, 10 s and 3.0 W, 20 s, both in contact and non-contact mode of laser beam delivery. Increases were only statistically significant in non-contact mode of laser irradiation (Table 2).

Temperature response curves of all recordings at the apically positioned thermocouple showed that temperatures were similar to that of the temperature of the surrounding water (37 °C).

### Scanning electron microscopy (SEM)

**Figure 3:**
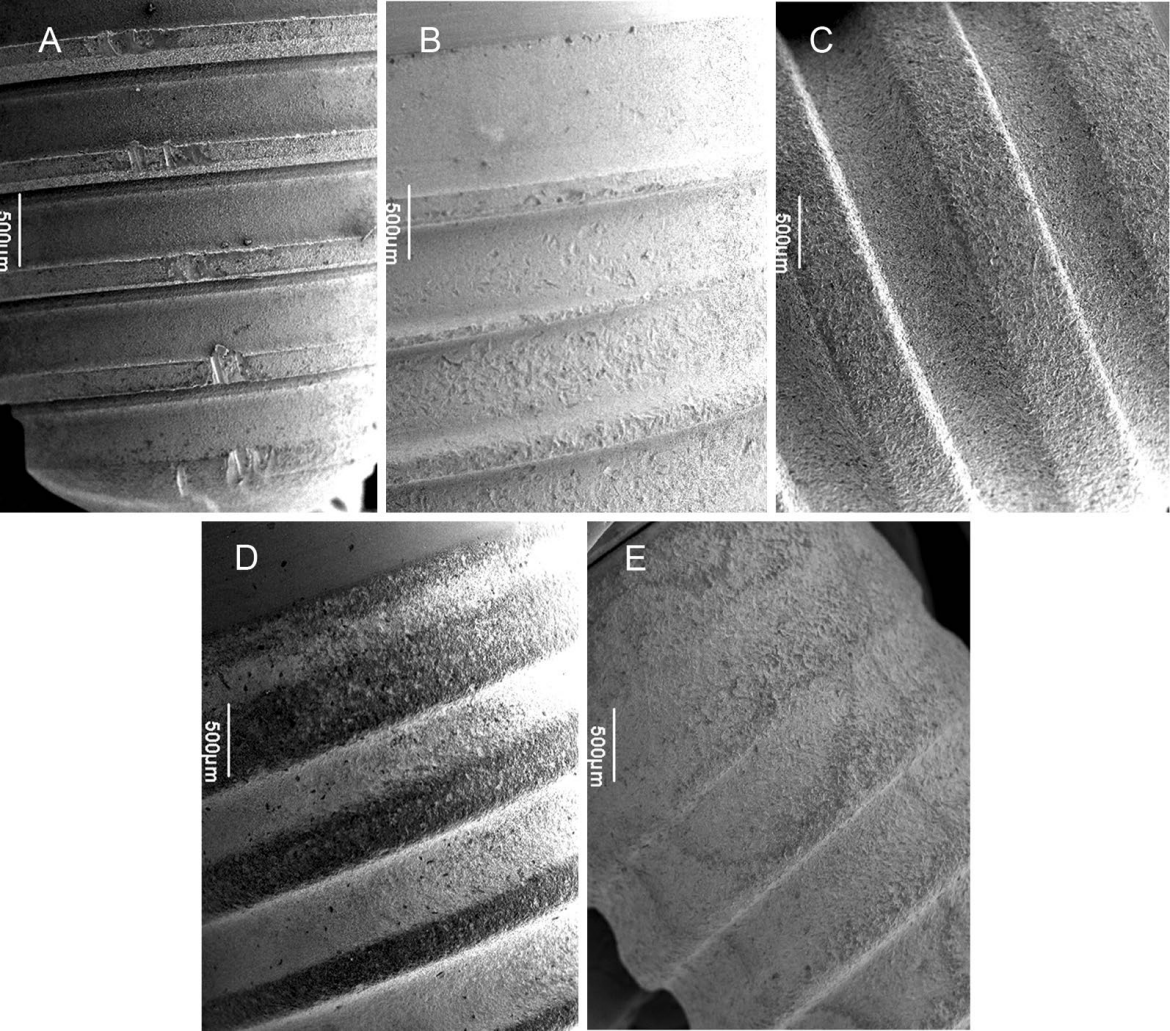
(**A**–**E**) Scanning electron microscopic aspects of the five dental implants following non-contact irradiation with continuous wave mode laser energy (0.8 W, 5 s): (**A**) NobelReplace Tapered Groovy RP, (**B**) Xive S plus, (**C**) Ankylos, (**D**) Straumann Pure ceramic, and (**E**) Straumann SLA Bone Level Roxolid. (*35-fold magnification*).

The areas of interest (AOI) surface sections of non-contact irradiated implants showed the same structure as control areas, with no signs of thermal damage, when parameters were chosen which had caused a thermal interface increase of less than 10 °C (Table 1). Similarly, the non-contact laser irradiation with the three parameter combinations showed no adverse results when the areas of interest were compared in the cervical, middle, and apical part of the implants. Accordingly, the three aforementioned parameters exert no adverse effects on the surface properties when irradiation is performed in non-contact mode, either on titanium or on ceramic surfaces (Fig. 3A–E).

## Discussion

Recent literature pointed out that the ineffectiveness of nonsurgical therapy of peri-implantitis is mainly attributable to the inefficient removal of biofilm from the complex implant surface^29^. To overcome this problem, various dental laser^1,23^ systems have been proposed for this purpose, including diode lasers. However, previous in vitro studies on temperature increases around dental implants have shown great discrepancies between different diode lasers^24^. Eriksson and coworkers found that increasing the temperature of bone tissue by ∆T 10 °C for 60 s causes permanent changes in the bone structure^26^. Accordingly, a tissue temperature gradient ΔT below 10 °C is stated as safe when limited to 60 s^1,15,17^.

**Figure 4:**
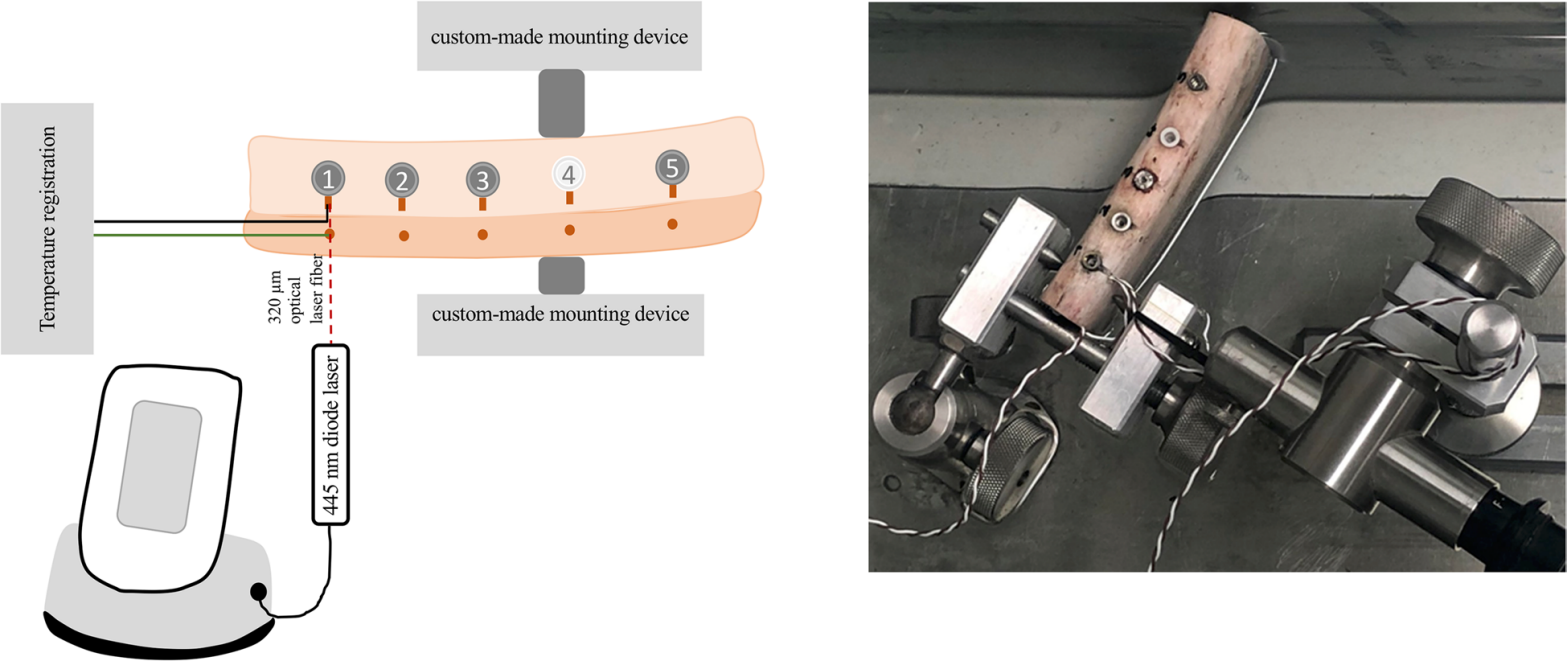
In vitro experimental setup. The implant-bearing bone is fixed in a water bath (37 °C). The 320 µm optical laser fiber of the SIROLaser Blue (Dentsply Sirona; Bensheim, Germany) aimed 90 degrees toward the dental implant surface in a horizontal direction. Two thermocouples were applied for temperature recordings on the side of laser irradiation: (**1**) at the implant–bone interface (black line) and (**2**) 3 mm above the most apical region of each implant (green line).

A blue-light semiconductor laser device with a wavelength of 445 nm was recently introduced for dental applications such as bacterial reduction^30^ and soft tissue surgery^31,32^. It was described that the lower laser energy absorption in surrounding soft tissues from scattering is expected to generate fewer thermal side effects^30^. However, it was unclear as to whether or not this would also translate into an advantage when the 445 nm wavelength is used for the decontamination process in the therapy of ailing implants. As a result of the experimental setup presented in this study, a total of 2880 temperature response curves were recorded. From a clinical point of view, only those parameter combinations which had caused a temperature increase of less than 10 °C were of interest. Accordingly, the presentation of results in Table 1 and Figs. 4–3 is limited to this clinically important threshold.

The present results indicate that the 445 nm laser wavelength is not inherently safe when used for the decontamination process of ailing implants. The use of the CW mode of laser irradiation, at a power of ≥ 0.8 W for ≥ 5 s, may cause deleterious temperature gradients to the surrounding marginal bone (Δ T ≥ 10 °C), which may occur following the application of both contact and non-contact modes of laser beam delivery. When working with the pulsed mode, the 50% duty cycle was only safe when the power was limited to ≤ 1.0 W and exposure times of ≤ 10 s in contact and non-contact mode of laser beam delivery. In contrast, the 10% duty cycle allowed the application of the maximum power output of 3 W even for 20 s, both in contact and non-contact mode.

Comparison of the present results with the literature is difficult due to limited data on this topic. Valente and coworkers^24^ used a similar sized implant (4 × 11 mm, Mk III, Nobelpharma, Yorba Linda; California, USA) and placed it in a small swine rib bone block. Two thermocouples were positioned at the contact interface between the bone and the implant, one crestally on the opposite side of the irradiated side, and another apically on the implant surface. This experimental setup was exposed to energy of three diode lasers (810 nm, 980, nm and 1,064 nm wavelengths). The implant was irradiated for 60 s, keeping the fiber optic tip of the laser 3 mm away from the implant surface, corresponding to the non-contact mode of laser irradiation in the present study. For irradiation, a power of 0.6 W, 0.8 W, and 1 W, was chosen, first in CW and then in pulsed mode. The authors stated that a critical increase of temperature of more than 10 °C was reached with all lasers at 0.8 W and 1 W in CW mode at room temperature. When the bone block was placed in a 37 °C water bath, a critical increase of temperature was not registered with any setting. The hottest peaks of temperature were always registered at the cervical point, which was the closest to the laser application, whereas at the apical thermocouple the values were within the safety range most times. The authors concluded that the use of these diode lasers does not cause a harmful increase in temperature when used under conditions similar to those of the human body. These results are only partially comparable to ours. Lengths and diameters of implants are in good accordance. Similarly to the findings of the aforementioned authors, it cannot be recommended to use 445 nm diode laser powers of ≥ 0.8 W in CW mode. However, the temperatures recorded were remarkably higher in the present study, even at a comparable power of 0.8 W and a shorter time of irradiation (5 s). It was intended to create a worst-case scenario; therefore, one thermocouple was positioned in the cervical interface 1 mm under the lasered area. In contrast, Valente et al. had placed their cervical thermocouple on the opposite side of the irradiation^24^. Therefore, it must be assumed that the results of the authors underestimate the thermal effect to a biologically significant extent.

In another recent study, Malmqvist et al.^1^ used the same diode laser device as in this study. In a similar setting using a pig mandible model, an Astra Tech OsseoSpeed EV 3.6 × 11 mm dental implant was placed in an edentulous area. Similarly, again, one of the thermocouples was placed next to the irradiation side. The laser handpiece was mounted on a stand and the optical fiber aimed 90 degrees toward the dental implant surface at 1 mm distance. A power setting of 1.0 W in CW mode was used for the 445 nm and the 970 nm diode laser wavelengths. With these parameters, a critical threshold of ∆T 10 °C was reached within a time period of only 2 s, which is in very good concordance with the present results (Fig. 1). When the implant was exposed to a power of 0.5 W in CW mode, an increase of ∆T 32 °C was recorded; application in pulsed mode (17% duty cycle 10 Hz, average 0.51 W) resulted in an increase of no less than ∆T 37 °C. Durations of laser irradiation were unfortunately not clearly defined, which hampers comparison with the present results. Interestingly, the authors assumed that the higher peak power with the pulsed mode led to a locally higher temperature than CW mode, which was not seen in the present study. The reasons are unclear; the present results are more in line with the literature pointing out that the pulsed mode of laser beam delivery reduces heat generation^14^.

Malmqvist and coworkers^1^ emphasized that soft tissue, saliva, and a blood flow through living tissue could further decrease the temperature in vivo. Moreover, they assumed that these cooling factors should put their in vitro results on the safe side when it comes to the clinical setting. In summary, the authors stated that using a 445 nm laser on dental implants is as safe as using a 970 nm laser in terms of temperature increase. However, this statement is inconclusive when regarding the results obtained (0.5 W in CW mode resulted in an increase of ∆T 32 °C). To the best of our knowledge, the literature has yet to show that cooling effects really exist when lasers are used for the decontamination process. Therefore, the conclusion drawn may only be appropriate when cooling the implant with water in a syringe. However, laser energy is absorbed, in part, in the cooling water and may not be sufficient to eliminate the biofilm. Moreover, the effective reduction of bacteria has been shown for the 810 nm diode laser in *S. sanguinis* or *P. gingivalis*, but not yet for the 445 nm diode laser^33^.

When looking at the present results in more detail, the five implant systems did not respond completely similarly to laser irradiation with respect to the thermal effect. In general, highest rises in temperature were seen in the Straumann Pure ceramic implant system. With respect to lowest increases, the Ankylos system was less at risk of exhibiting critical marginal temperatures at the interface than the other three metal systems. Comparison of these results with those of the literature is crucial because the available literature has not yet focused on these topics.

The results of this in vitro study indicate that the 445 nm laser irradiation may have adverse thermal effects on the surrounding bone of lasered implants. Consequently, 445 nm laser irradiation parameters have to be chosen carefully for the purpose of peri-implant care. In order to understand the results, materials of the dental implants must be taken into consideration. Because of the weak thermal conductivity of titanium (λ_Ti_ = 22 W/mK, λ_Au_ = 316 W/mK), the generated heat dissipates relatively slowly, and therefore can accumulate. For zirconium oxide, an even weaker thermal conductivity of λ_ZrO_ = 3 W/mK is reported^34^, which explains the higher temperatures recorded at the bone–implant interface. Accordingly, heat accumulates in the ceramic material and dissipates to a lesser extent as compared to titanium. Due to the weaker thermal conductivity of 0.68 W/mK in human cortical bone and 0.42 W/mK in human bone marrow^35^, it remains questionable whether cooling effects can really occur in clinical settings.

Moreover, implant geometry should also be taken into consideration when discussing thermal effects on laser irradiated implants. When irradiating different titanium implants, thicker walls will absorb more energy compared to thinner ones. In addition, the specific thermal capacities of irradiated materials are known to have an impact on temperature response curves. The specific thermal capacity of titanium (c _Ti_ = 523 J/kg K)^34^ is higher than that of zirconium oxide (c _ZrO2_ = 418 J/kg K)^34^, which means that the latter material needs less energy to heat up. Therefore, clinicians ought to be aware that different dental implant materials and geometries show different temperature response curves when subjected to 445 nm diode laser energy.

With respect to SEM findings, Malmqvist et al.^1^ found no surface alterations for the 445 nm wavelength when radiating for 4 min at 2.0 W with a distance of 1 mm. Also in the present study, non-contact irradiation did not alter implant surfaces, either following CW mode of laser beam delivery (0.8 W, 5 s) or pulsed mode of laser beam delivery (10% duty cycle, 3 W, 20 s, and 50% duty cycle, 1 W, 10 s). In contrast, preliminary trials have shown that CW mode laser irradiation at 0.8 W for 5 s in contact mode resulted in macroscopically visible black staining of the titanium surfaces. Therefore, these surfaces did not undergo evaluation in more detail; all further irradiations were performed in non-contact mode. Further studies need to evaluate surface damage following CW mode laser irradiation.

In another recent study, Scarano and coworkers^2^ observed the detoxifying effect of Er-YAG laser on implant surfaces retrieved from patients affected by peri-implantitis. Therefore, a total of 45 implants, which had been removed for peri-implantitis reasons, were divided into 2 groups: group I 22 nonirradiated implants; group II 23 irradiated implants. Irradiation was performed with an erbium laser (variable pulse frequency (20 pps), pulse energy 75 mJ). The fiber tip was guided in a circular motion from coronal to apical parallel to the implant surface in contact mode. The implant surfaces were examined under a Scanning Electron Microscopy operating at 20 to 30 kV with tilt angles ranging from 10 to 45 degrees. Due to the results obtained by SEM, the authors found the Er-YAG laser highly efficient and effective in removing contaminants from the implant body without overheating or altering the surface characteristics of the roughened titanium surface. Accordingly, they concluded that the absence of any measurable changes to the titanium surface and the lack of an organic smear layer create the ideal environment for the regrowth of bone and potential reintegration of the exposed or contaminated area of the implant body. These results are in line with the findings of the present study. Although the present study did not include contaminated implants, the respective SEM results indicated absence of surface alterations following 445 nm laser irradiation when used in non-contact mode of laser beam delivery.

This study has several limitations, because the efficacy of 445 nm laser irradiation for removal of bacterial microbiota was not tested. In addition, morphological surface analysis was performed with second electron (SE) scanning electron microscopy (SEM), not with backscattered electrons (BSE). The latter technique provides high-resolution images of the elements present within in a sample. In contrast, secondary electrons originate from the surface or the near-surface regions of the sample. To the knowledge of the authors it seems unlikely that fluencies used in this experimental setting will result in chemical or morphological alterations within the implant specimen; however, this cannot be excluded, and therefore, needs to be investigated in a further study. Finally, chemical surface analysis was not performed. Again, also chemical alterations of the irradiated implants seem to be unlikely due to the fluencies. Nevertheless, chemical characterization of the irradiated implant surfaces with X-ray photoelectron spectroscopy (XPS) will be necessary in further studies.

## Methods

### Dental laser

The diode laser employed was the model SIROLaser Blue manufactured by Dentsply Sirona (Bensheim, Germany) (Fig. 4). This system provides three different laser wavelengths with different power output ranges and can be operated in either a CW or pulsed mode of laser beam delivery: λ_1_ = 445 ± 5 nm, P (CW) = 0.2–3.0 W, λ_2_ = 970 -10/ + 15 nm, P (CW) = 0.2–2.0 W and λ_3_ = 660 ± 5 nm, P (CW) = 25, 50, 100 mW. The CW mode implies a continuous, uninterrupted laser beam as long as the laser is activated (and determined by a time set). Moreover, a chopped mode is provided, in literature sometimes referred to as "pulsed mode". In this mode, the laser beam is interrupted at regular intervals, which can be adjusted via the duty cycle [%]. The duty cycle is defined as the ratio between the pulse duration (the duration that the laser beam is actually activated) and the total period of time (which is the time from the beginning of a pulse to the beginning of the next pulse)^36^. In the pulsed (chopped) emission mode, the system allows pulse duration between 10 μs and 0.99 s; i.e. 1 Hz to 10 kHz. This rate is the modulation frequency of the laser unit. The average power is the product of power release and duty cycle. When a power release of 3 W is used with a duty cycle of 10% (50%), the average power is 0.3 W (1.5 W). Moreover, when a power of 3 W (1 W) is used with the 10% (50%) duty cycle for a total of 20 s (10 s), an energy of 6 J (5 J) is applied. When working with a power emission of 3.0 W, a frequency of 100 Hz is delivered by the laser device^36^.

Laser light is transmitted at all three wavelengths by flexible quartz glass fibers of 320 µm or 200 µm core diameter (coating diameters 408 µm and 270 µm, respectively). According to the manufacturer, all diameters may differ ± 20%^36^. In the present study, a 200 µm core diameter fiber was provided. An in-house-measurement with use of a light microscope (Zeiss Primo Star, D—Jena) verified a core diameter of 220 µm. The 445 nm wavelength laser is classified as Class IV, the 660 nm wavelength laser as Class IIM (DIN EN 60,825-1: 2015-07). In this study, thermal effects of the 445 nm laser beam were evaluated using an in vitro model for the purpose of implant surface decontamination. Therefore, power, wattage, and exposure time of the laser device were varied according to the experimental in vitro setup.

The amount of energy delivered by this device was measured before and following each irradiation with use of the integrated calibration system (Fig. 4). This procedure was performed to guarantee constant energy density.

### Dental implant systems

For this study, a total of ten brand-new dental implants of five commercial systems were available (system, diameter x length), two implants of each system: Ankylos 4.5 × 9.5 mm, Xive S plus 4.5 × 13 mm (both systems Dentsply Sirona; Mannheim, Germany), NobelReplace Tapered Groovy RP 4.3 × 10 mm (Nobel Biocare; Gothenburg, Sweden), Straumann SLA Bone Level Roxolid 4.1 × 10 mm and Straumann Pure 4.1 × 10 mm (both systems Straumann AG; Freiburg, Germany). These systems differ with respect to their material and surface modifications, which have a major impact on the absorption of laser light. Therefore, the implant characteristics are presented in detail below, according to information of the manufacturers.

The Ankylos implant was made of commercially pure titanium grade 2. Surface modification was yielded via large grit blasting (Al_2_O_3_ particles 354–500 μm) and high-temperature acid etching. The latter results in micro-pits of 0.5–1 μm and an average micro-roughness of 1.40–1.75 μm. The Xive S plus implant was also made of commercially pure titanium grade 2. The Friadent plus Surface consisted of horizontal threads and a high-temperature acid-etched micro-structure which results in a micro-roughness of > 2 μm. The NobelReplace Tapered Groovy RP implant consisted of commercially pure titanium grade 4. The TiUnite surface was characterized as a thickened titanium oxide layer which was developed by anodization in a phosphoric electrolyte. This process generated a porous surface with a micro-roughness of 1–2 μm. The Straumann bone level implant was made of Roxolid, an alloy consisting of 15% zirconium and 85% titanium. The SLA surface showed a macro-roughness of 20–40 μm peak-to-peak and a micro-roughness of 2–4 μm. Surface modification was performed with the use of large grit (250–500 μm) blasting and acid etching (HCL/H_2_SO_4_). In contrast to these four metal dental implants, the Straumann Pure implant consisted of yttrium-stabilized zirconium oxide (Y-TZP). Its Straumann ZLA surface is characterized by a macro- and micro-roughness which is very similar to the topography of the SLA surface.

### Measurement of thermal effects

In this study, an “ailing” implant decontamination protocol was simulated. For this purpose, in line with the literature, a rib of a recently slaughtered pig, intended for consumption, was obtained from a butcher^25^. The soft tissues of the rib were cut off. To allow comparison with the human alveolar process, the rib was segmented and only the inferior part was used for implant installation. Moreover, edges of the rib were sealed with silicone (Optosil, Kulzer, D-Hanau). Ethical approval was not required for this in vitro study. Each implant cavity preparation and implant insertion was performed by one surgeon (HD) and standardized according to each implant system manufacturer’s protocol. Surgery was performed with a standard implant surgery device (Implant Center 2, Acteon; Mettman, Germany). The position of the implant systems in the bone was chosen randomly. Bony cavities were prepared at 800 rpm with continuous cooling with 0.9% NaCl solution with the aforementioned diameters and lengths. Accordingly, all five bony cavities allowed complete insertion of fixtures on the bone level.

A marginal defect, 2 mm in diameter, was drilled at each marginal rim of the implant cavities with a steel bur to simulate peri-implantitis induced bony defects in the collar region of the implants which is the zone of the implant that normally is in touch with the soft tissues (Fig. 4).

When an implant was inserted in its bony cavity, the end of a temperature probe (CrNi-Mo type T thermocouple XF-1095-FAR, Labfacility Limited. Temperature and Process Technology, Sheffield, United Kingdom) was held simultaneously in the interface between implant body and bone, directly under the deepest rim of the simulated 2 mm peri-implantitis—induced bony defect. Thereby, the temperature probe was stabilized by the inserted implant.

The implant-bearing bone was fixed in a custom-made mounting device and positioned in a 37 °C water bath (VWB 2, VWR International GmbH; Darmstadt, Germany). The quartz glass fiber (core diameter 220 µm) was positioned with use of a custom made mounting device in the center of the simulated 2 mm peri-implantitis—induced marginal bony defect. Accordingly, the distance between fiber and marginal bone measured [2000 µm minus 220 µm]/2 = 890 µm. Therefore, in a vertical direction, the lased zone ended nearly 1 mm above the marginal rim of the bone, adjacent to the fixed thermocouple. In horizontal direction, the optical laser fiber was fixed either in direct contact with the implant surface or in a non-contact position with 2 mm distance from the implant surface^17^. In this setup, the optical fiber aimed 90 degrees toward the dental implant surface in a horizontal direction^1^.

According to this experimental set up, the laser beam measured 220 µm in diameter when positioned in direct contact with the implant surface. Similarly, due to the collimation of laser radiation, the laser beam diameter was also 220 µm after the 2 mm distance in the non-contact position.

Care was taken to ensure that the marginal peri-implant defect zone was not immersed. This prevented the water from dissipating the heat generated by the laser at the cervical aspect of the implant, which might have compromised the results.

In addition, another hole drilled 3 mm above the most apical region of the five implant cavities allowed the positioning of a second thermocouple. Both thermocouples (cervical, apical) were connected to a thermoelementlogger (GSI Systementwicklung und Instrumentierung GmbH; Aachen, Germany) that recorded the temperature increases.

The model system was stabilized at room temperature for 12 h prior to beginning the irradiation procedure. Temperature changes were recorded using a personal computer connected to the thermocouples. Temperatures were registered every second starting a few seconds before the start of the tests and continuing at least 2 min after the laser was turned off for each respective test.

Thermal effects of 445 nm laser irradiation were tested in both CW and pulsed mode with different levels of power and exposure times (0–3.0 W and 5, 10, 15, and 20 s). With a maximum power output of 3 W, the fiber of 320 µm in diameter (core diameter 220 µm, surface area without cladding 3.8 × 10^–4^ cm^2^) permits delivery of a mean power density of E = 7894.74 Wcm^−2^. For example, with an irradiation time of 10 s in CW mode, an energy fluence of H = 78,947 J cm^−2^ is applied at a tip angle set at 90 degrees. The experimental design allowed evaluation of the interaction between laser wattage output and duration of exposure time: (1) CW in contact/non-contact, each at eight power levels (0.2, 0.6, 0.8, 1.0, 1.6, 2.0, 2.6, 3.0 W) and four exposure times (5, 10, 15, and 20 s), (2) pulsed mode in contact/non-contact, each at the aforementioned eight power levels and four exposure times, each at 10% and 50% duty cycle. Before measurements started, calibration of the laser power was performed using the internal calibration system of this laser device. Parameter combinations (1) and (2) were tested on all five implant systems. Moreover, at each step, the temperature at the end of the exposure time was recorded. Finally, the mean temperature of three measurements was calculated. In summary, temperature rise response curves of a total of 2880 different combinations of parameters were recorded.

### Scanning electron microscopy

In part two of the in vitro study, SEM was performed. The SEM analyses were performed with a JEOL scanning electron microscope (JEOL Inc.; Peabody, MA, USA) type ISM-35CF with an accelerating voltage of 10.0 kV, a probe current of 0.13 nA, and focus distance of 5.0 mm. The surface of the implants was sputtered homogeneously with a layer of gold of 3 nm in thickness following laser irradiation. Another five brand-new dental implants of the aforementioned five commercial systems were used for this purpose. The implants were removed from their containers and fixed on a custom-made mounting device. The aforementioned optical laser fiber was fixed in a non-contact position with 2 mm distance from the implant surface^17^. In this setup, the optical fiber aimed 90 degrees toward the dental implant surface in a horizontal direction^1^. Along the whole implant length, three standardized areas of interest (AOI) were defined (cervical, middle, and apical part of the implant). The cylindrical shape of the implants allowed the definition of another six (2 × 3) AOI on the two lateral aspects of the aforementioned areas (circumferential position 3 × 120°). Areas of interest were lasered at those three power levels which had caused a rise in temperature up to a maximum of 10 °C (Table 1). Accordingly, each implant was irradiated on the cervical, middle, and apical part with the three aforementioned power levels. This resulted in nine irradiations (3 × 3) per implant. The procedure allowed a comparison of the effects of the three laser parameters on the cervical, middle, and apical part of each implant. Adjacent nonirradiated areas served as controls. Findings were documented on film (FP 4, 125 ASA). Two images at two different magnifications (35-fold and 100-fold) were taken from each of the nine AOI of each implant.

### Statistical analysis.

The data were analyzed by means of Statistical Package for Social Sciences program (SPSS 19.0, Chicago III, USA). Data of the temperature increases are presented as means ± standard deviation (SD). Statistical testing comparing mean of two implant systems for temperature increases was performed using Student’s t test. A two-sided P-value less than 0.05 was considered to indicate statistical significance.

## Conclusion

Within the limits of this study it must be stated that the 445 nm diode laser is not inherently safe for the decontamination of ailing implants. Laser irradiation in continuous wave (CW) mode (≥ 0.8 W), contact mode of laser beam delivery, and higher power outputs (≥ 1.0 W) in pulsed mode for longer exposure times (> 10 s) may cause harmful rises in temperature of more than 10° C in the interface. However, both CW and pulsed laser irradiation can prevent harmful rises in temperature when used at moderate parameters (1 W, 10 s, 50% DC or 3 W, 20 s, 10% DC). With use of scanning electron microcopy, no surface alterations were seen on the tested implant systems when using these parameters. With respect to the tested implant systems, the Straumann Pure ceramic implant system was more at risk to overheat the marginal interface zone as compared to titanium implants, both in contact and non-contact mode. Among the tested titanium implants, the Ankylos system showed lowest marginal temperature increases in almost all parameter combinations. From these results it was concluded that different dental implant materials and geometries show different temperature response curves when subjected to 445 nm diode laser energy. Therefore, manufacturers of laser devices should provide implant-specific laser parameters for the decontamination process. Further studies are necessary to capture the cross-section morphology and chemistry of implants following laser irradiation and to evaluate the bactericidal effects of the 445 nm diode laser energy on contaminated dental implant materials.

## Data Availability

A total of 2,880 temperature response curves were recorded. From a clinical point of view, only those parameter combinations which had caused a temperature increase of less than ∆T 10 °C were of interest. Accordingly, the presentation of results in Tables 1, 2 and Figs. 4–3 is limited to this clinically important threshold. If needed, results are available from the first author (HD).
